# Average Throughput Performance of Myopic Policy in Energy Harvesting Wireless Sensor Networks

**DOI:** 10.3390/s17102206

**Published:** 2017-09-26

**Authors:** Omer Melih Gul, Mubeccel Demirekler

**Affiliations:** Department of Electrical and Electronics Engineering, Middle East Technical University (METU), 06531 Cankaya, Ankara, Turkey; demirek@metu.edu.tr

**Keywords:** energy harvesting, decision making, resource allocation, scheduling policy, wireless sensor network

## Abstract

This paper considers a single-hop wireless sensor network where a fusion center collects data from *M* energy harvesting wireless sensors. The harvested energy is stored losslessly in an infinite-capacity battery at each sensor. In each time slot, the fusion center schedules *K* sensors for data transmission over *K* orthogonal channels. The fusion center does not have direct knowledge on the battery states of sensors, or the statistics of their energy harvesting processes. The fusion center only has information of the outcomes of previous transmission attempts. It is assumed that the sensors are data backlogged, there is no battery leakage and the communication is error-free. An energy harvesting sensor can transmit data to the fusion center whenever being scheduled only if it has enough energy for data transmission. We investigate average throughput of Round-Robin type myopic policy both analytically and numerically under an average reward (throughput) criterion. We show that Round-Robin type myopic policy achieves optimality for some class of energy harvesting processes although it is suboptimal for a broad class of energy harvesting processes.

## 1. Introduction

### 1.1. Motivation

The Internet of Things (IoT) is an intelligent large-scale communication infrastructure of uniquely identifiable devices capable of communicating with each other wirelessly through the Internet [1]. The devices in an IoT structure are typically equipped with wireless sensors [2]. Wireless Sensor Networks (WSNs) provide the opportunity of efficient data collection and transmission anywhere [3]. Thus, WSNs have various applications, such as agriculture [4], ambient air monitoring [5,6], frost monitoring [7], structural health monitoring [8,9,10], remote assistance for elderly people [11], home monitoring [3,11,12,13] and smart cities [14,15]. Being frugal with energy consumption is important to several WSN deployments. Energy harvesting (EH) [16] can particularly facilitate WSN applications where replacing battery is not practical. Therefore, energy harvesting is a promising approach for the emerging IoT technology [17]. Energy may be harvested from the environment in several different ways (solar, piezoelectric, wind, etc.) [17]. As energy harvesters generally depend on uncontrollable energy resources and the amount of harvested energy is generally low [17,18], WSNs need robust, self-adaptive, energy efficient policies to optimize their reliable operation lifetime [19,20].

In this paper, we consider a fusion center (FC) collecting data from *M* EH wireless sensors. At each time slot (TS), *K* sensors are scheduled for data transmission by the FC, which does not have the direct knowledge of the battery states of the sensors or the statistics of their EH processes. It is assumed that the communication is error-free and the sensors are data backlogged but limited in available energy. Each sensor has an infinite-capacity battery to store the harvested energy and battery leakage is ignored. When a sensor is scheduled in TS *t*, it sends data to the FC in the TS *t* as long as it has enough energy in that TS. Sending one packet takes up one TS. The objective of the FC is to maximize the average throughput over a time horizon.

In fact, battery states can be made available to the FC through some additional cost (i.e., feedback) and complexity in some WSNs. However, sending information about the battery state will cause extra time and energy consumption, which we avoid. Assume that the header containing only battery state is *H* bytes and the remaining part (payload + other headers) of the data packet is *P* bytes, then sending information about battery state will cause $\frac{H}{P}$ times more time and energy consumption than those consumption not sending no information about battery state. We can avoid extra time consumption by consuming significantly more energy. As it is well known in communication field, data transmission rate is a concave function of transmission power. In fact, the well known Shannon’s capacity formula [21] ( Shannon’s capacity formula is $C=B\log_{2}\left(1+\frac{S}{N}\right),$ where *C* is the maximum capacity of the channel in bits/second otherwise called Shannon’s capacity limit for the given channel, *B* is the bandwidth of the channel in Hertz, *S* is the signal power in Watts and *N* is the noise power, also in Watts. The ratio $\frac{S}{N}$ is called Signal to Noise Ratio (SNR)) indicates that this concave function is a logarithmic function. Therefore, when sending info about battery state, we can avoid the extra time consumption only by consuming much more extra energy than $\frac{H}{P}$. For example, assume that the overhead containing knowledge of battery state is one fourth of the exact data, i.e., $\frac{H}{P}=\frac{1}{4}$. Then, sending both overhead and exact data instead of sending only exact data in the same time duration may cause two times more energy consumption. Thus, it can be said that although energy consumption and network lifetime are not performance metrics in the problem at hand, the problem definition (with sending no information about battery states) helps sensors decrease energy consumption per data packet transmission and thus network lifetime can be increased. Therefore, it is more relevant from a practical perspective that the FC makes scheduling decisions without any knowledge about battery states or statistics of their EH processes [22].

To set up the problem, a model about generation and usage of energy is needed. Each sensor accesses the energy state of its own battery only at the beginning of the time slots in which it is scheduled by the FC. Moreover, independent from the functional form (linear or other) and type of energy harvesting resource (solar, wind, piezoelectric, RF, etc.), the net amount of harvested energy minus used energy is stored losslessly. This assumption is consistent with typical batteries in use today for which leakage is negligibly small over several minutes because battery leakage causes the battery to self-discharge less than 10% (10% for Nickel-based batteries and 5% for Lithium-ion batteries) in 24-h from the results in [23]. Based on these mild assumptions about EH processes, an appropriate performance criterion is the average throughput (reward) criterion over a time horizon rather than expected discounted throughput (reward) for the problem at hand [24].

### 1.2. Related Work

Although EH processes are not limited to be Markovian in this work, under Markovian assumption, the problem could be formulated as a partially observable Markov decision process (POMDP) [25]. In this case, dynamic programming (DP) [26] may be employed for optimal solution. However, DP has exponential complexity, which limits its scalability [27].

A second approach is reinforcement learning by considering the problem as a POMDP. Q-learning [28], one of the most effective model-free reinforcement learning algorithms, would guarantee convergence to an optimal solution in this problem. However, its very slow convergence [29] deems it non-ideal for a problem with a sizeable state space, especially as the discount factor approaches 1. R-learning [30], which maximizes the average reward, may be considered; however, there is no guarantee on the convergence of R-learning. Therefore, reinforcement learning do not seem to be suitable for obtaining an efficient solution to this problem. There are other approaches that can, in the long run, guarantee convergence to optimal behavior. However, in many practical applications, a policy that achieves near optimality very quickly is preferable to the one that converges too slowly to exact optimality [29].

Another approach to this problem is to set it up as a restless multi-armed bandit (RMAB) problem. An optimal solution was proposed for RMAB problem under certain assumptions by Whittle [31]. It is shown that finding the optimal solution to a general RMAB problem is PSPACE-hard [32] (In complexity theory, PSPACE is the set of all decision problems that can be solved by a Turing machine using a polynomial amount of space). As a policy with a reasonable complexity, myopic policy (MP) has been suggested for various RMAB problems. While MP is not optimal in general since it focuses only on the present state [33], it can be proven to be optimal in certain special cases.

A very similar problem to the problem at hand is investigated in [34,35]. In fact, we pose the same problem in [34,35] with the exception that we assume infinite capacity battery without leakage at the sensors, in contrast to [34,35] where either no battery or unit capacity batteries with leakage are assumed. Both [34,35] formulate the problem as a POMDP, and, due to the myopic approach in these work, the focus is on the immediate reward instead of future rewards. In [35], a single-hop WSN consisting of EH sensors with unit capacity batteries (i.e., able to store only one transmissions’s worth of energy) and a fusion center is posed as a RMAB problem. The optimality of a round-robin (RR) based MP is proved under certain specific assumptions. Then, it is shown that this RR based MP coincides with the Whittle index policy, which is generally suboptimal for RMAB problems [36], for a specific case. In [34], the problem is formulated as a POMDP and the optimality of a MP is proven for two cases: (1) the sensors are unable to harvest and transmit simultaneously, and transition probabilities of the EH processes are affected by the scheduling decisions, and (2) the sensors have no batteries.

In [37,38,39], we investigate quite a similar problem with the problem at hand, although the problem in [38] has some differences due to its system model. In this paper, we consider more general class of energy harvesting processes than [37,39] do (as it is explained in the rest of this paper, we consider energy harvesting processes with intensities both ρ≤1 and ρ>1 in this paper, whereas we consider only energy harvesting processes with intensities ρ≤1 in [37,38,39]. Besides this, in this paper, we also consider the cases for which finding exact throughput performance of the myopic policy is not possible with using only intensities. For these cases, we find an upper bound for the throughput performance of the myopic policy).

### 1.3. Our Contributions

Main contributions of the paper are summarized as follows:

- The EH WSN problem is studied under average throughput (reward) criterion and no battery leakage assumption for the most general class of EH processes whereas the problem is studied under discounted throughput (reward) criterion and battery leakage assumption for certain specific cases in [34,35].
- This paper considers a battery capacity (infinite-capacity) larger than unit capacity (which is the maximum battery capacity considered in [34,35]) for the EH WSN problem.
- We show that under average throughput criterion and infinite-capacity battery assumption, RR policies including the MP in [34,35] achieve optimality for some class of EH processes although they are suboptimal for a broad class of EH processes.
- We obtain an upper bound for throughput performance of the RR policies under average throughput criterion for quite general (Markov, i.i.d., nonuniform, uniform, etc.) EH processes. Furthermore, we show that all RR policies including the myopic policy achieve almost the same throughput performance under an average throughput criterion.
- Compared with [34,35], we consider more reasonable finite capacity battery case in the numerical results and show that there is a slight difference in throughput performance between the finite capacity battery case and infinite capacity battery case.

### 1.4. Organization of the Paper

The rest of this paper is organized as follows. The system model and problem formulation are given in Section 2. In Section 3, we show that RR based MP in [34,35] cannot achieve 100% throughput for a broad class of EH processes under average throughput (reward) criterion. Moreover, we obtain an upper bound for throughput performance of RR policies including the myopic policy under average throughput criterion. Furthermore, we show that RR policies including the myopic policy achieve almost the same throughput as each other. In Section 4, numerical results show that the myopic policy is suboptimal for a broad class of EH processes, which supports the results found in Section 3. Section 5 concludes the paper and provides some future directions.

## 2. System Model and Problem Formulation

**Figure 1:**
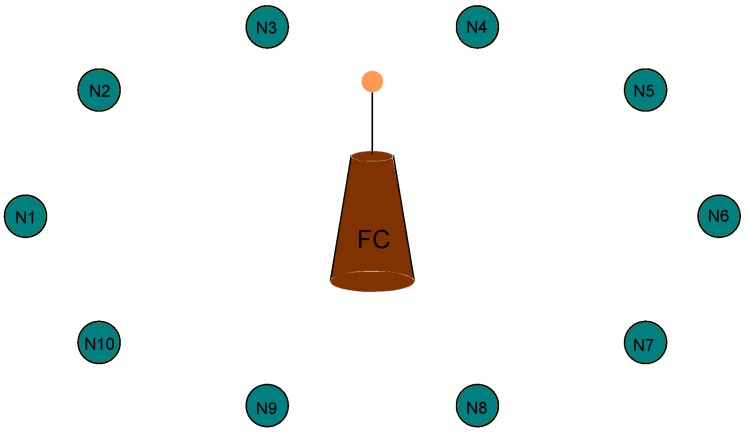
An example single hop WSN where an FC collects data from 10 EH sensors.

We consider a single-hop WSN where a fusion center (FC) collects data from *M* EH-capable sensors (please see Figure 1). The index set of all sensors is denoted by $S=\left\{1,2,\ldots ,M\right\}$. The WSN operates in a time-slotted fashion indexed as t=1,2,…,T. At the beginning of each TS, the FC schedules *K* sensors for data transmission by assigning each sensor to one of its *K* mutually orthogonal channels. As the research community working on multi-channel protocols generally either assume that channels are perfectly orthogonal (interference-free) or consider the use of only orthogonal channels [40], we assume that the channels are mutually orthogonal, i.e., there is no interference. If the sensors send data at a low data transmission rate and interference management is applied, very low-error transmission can be achieved. Therefore, we assume error-free transmission in the WSN. We assume that the sensors always have data to send as it is assumed in [34,35]. When you consider a single hop wireless sensor network in a wide lowland (flat cropland), there will be no obstacles like buildings, hills which may cause shadowing, reflection, refraction or absorption/diffractions, etc. In a single hop WSN with a central scheduler, the sensors are expected to send the same type of data such as humidity, temperature, pressure, etc. Considering the applications of WSNs in agriculture and frost monitoring [41,42,43,44,45], the sensors have nearly the same propagation conditions to send the same type of data in large croplands. Therefore, we assume that data packets have equal size and sending one packet takes up one TS. A *unit energy* is defined as the energy required for a sensor to send one packet in one TS.

The energy harvested by sensor *i* in TSs 1 through *t* is denoted by $E_{i}\left(t\right)$, and the energy harvested in TS *t* is denoted by $E_{i}^{h}\left(t\right)$; i.e., $E_{i}^{h}\left(t\right)=E_{i}\left(t+1\right)-E_{i}\left(t\right).$ For t=1,…,T, we define the *activation set*, denoted by π(t), as set of the sensors scheduled in TS *t* under a policy π.

As it is assumed in [34,35], if a sensor has sufficient energy and scheduled in TS *t*, it sends one data packet to the FC in TS *t*. The number of data packets sent by sensor *i* in TS *t* under a policy π can be written as $I_{\left\{i\in \pi (t)\right\}}I_{\left\{B_{i}^{\pi }\left(t\right)\geq 1\right\}}\in \left\{0,1\right\},$ where $I_{\left\{.\right\}}$ is the indicator function and $B_{i}^{\pi }\left(t\right)$ is the stored energy in infinite-capacity battery of sensor *i* in TS *t* under a policy π. Under the policy π, $B_{i}^{\pi }\left(t\right)$ is evolved as

(1) $$B_{i}^{\pi }\left(t+1\right)=B_{i}^{\pi }\left(t\right)+E_{i}^{h}\left(t\right)-I_{\left\{i\in \pi (t)\right\}}I_{\left\{B_{i}^{\pi }\left(t\right)\geq 1\right\}}\;\forall i,t.$$

The number of data packets sent by all sensors to the FC within the first *t* TSs under a policy π is denoted by $V^{\pi }\left(t\right),$ which is $V^{\pi }\left(t\right)=\sum_{i=1}^{M}V_{i}^{\pi }\left(t\right),$ where the number of packets sent by sensor *i* in TSs 1 through *t* under a policy π is denoted by $V_{i}^{\pi }\left(t\right)=\sum_{\tau =1}^{t}I_{\left\{i\in \pi (\tau )\right\}}I_{\left\{B_{i}^{\pi }\left(\tau \right)\geq 1\right\}}$.

In [34,35], the objective is to find a policy that maximizes the total throughput over the time horizon under expected discounted reward criteria, where the discount factor corresponds to battery leakage. (Ref. [34] considers the problem under discounted throughput (reward) criteria since [34] assume battery leakage with discount factor 0.9 such that stored energy decreases to 90% in a time slot which is generally less than 1 ms. However, this is not realistic with recent battery technology.) On the other hand, battery leakage in typical batteries causes less than 10% decrease in the stored energy in 24 h [23]. This decrease implies that battery leakage in a 1 ms-long time slot is less than 0.000000005% and so the discount factor is greater than 0.9999999988, i.e., 0.9999999988≤β<1 (Twenty-four hours equals to 86400000 ms. If length of a time slot is chosen as 1 ms, then $0.90\leq \beta^{86400000}<1$ which implies that 0.9999999988≤β<1). Therefore, we neglect battery leakage in our problem formulation, which is practical in terms of engineering aspects. As the problem at hand assumes infinite data backlog and no battery leakage from [19,23], it is delay insensitive by nature. Hence, from [19,23,24], we formulate the scheduling problem as follows.

**Problem** **1.** *Average throughput (reward) maximization over a time horizon, T*

$$\begin{array}{cc} \underset{{\left\{\pi (t)\right\}}_{t=1}^{T}}{max} & \frac{1}{T}V^{\pi }\left(T\right),\\ s.t. & Label\;(1). \end{array}$$

The following notions are used in the rest of the paper.

**Definition** **1.** *For a given sequence of energy harvests, an* ***optimal policy***, $\pi^{*}$, *is a policy that maximizes the total throughput of all sensors upto* KT *over a time horizon, T, i.e.*, $\pi^{*}≜\arg\max_{\pi \in G}\frac{V^{\pi }\left(T\right)}{T},$ *where G is set of feasible policies.*

**Definition** **2.** *A* **fully efficient policy**, $\pi^{FE}$, is a policy under which the sensors use up all of their harvested energy at the end of the time horizon which yields $V^{FE}\left(T\right)=\sum_{i=1}^{M}V_{i}^{FE}\left(T\right)$ (Although we use $V^{\pi }\left(t\right)$ to denote the total throughput achieved in first t TSs under a policy π, the total throughput achieved in first t TSs under a policy $\pi^{FE}$ is denoted by $V^{FE}\left(T\right)$ instead of $V^{\pi^{FE}}\left(T\right)$ for simplicity. Moreover, $V^{*}\left(T\right)$ and $V^{RR}\left(T\right)$ are used instead of $V^{\pi^{*}}\left(T\right)$ and $V^{\pi^{RR}}\left(T\right)$, respectively. Similarly, $V_{i}^{FE}\left(T\right)$, $V_{i}^{*}\left(T\right)$ and $V_{i}^{RR}\left(T\right)$ are used instead of $V_{i}^{\pi^{FE}}\left(T\right)$, $V_{i}^{\pi^{*}}\left(T\right)$ and $V_{i}^{\pi^{RR}}\left(T\right)$, respectively.), where $V_{i}^{FE}\left(T\right)=\left\lfloor E_{i}\left(T\right)\right\rfloor$.

For certain EH processes, an optimal policy may not be a fully efficient policy, as it is explained in Remark 1.

**Definition** **3.** ***Efficiency*** *of a policy π, denoted by η(π), is defined as the ratio of the throughput of a policy π over the throughput of a fully efficient policy, $\pi^{FE}$, over the time horizon, T. It can be expressed as*

(2) $$\eta \left(\pi \right)≜\frac{V^{\pi }\left(T\right)}{V^{FE}\left(T\right)},$$

*where $V^{\pi }\left(T\right)$ and $V^{FE}\left(T\right)$ are the number of collected data packets (throughput) over a time horizon T under a policy π, and fully efficient policy $\pi^{FE}$, respectively (When K and T are in order of tens and thousands, respectively, throughput of an optimal policy is expected to be in the order of ten thousands. The term, efficiency, provides us the opportunity of dealing with small numbers less than or equal to 1 instead of large throughput numbers. Efficiency of a policy also gives us the relative throughput of that policy to the throughput of a fully efficient policy, which provide convenience in numerical results).*

The efficiency term itself can also be considered as relative energy consumption of the system to total energy harvested by the system.

The number of data packets which can be sent by all sensors from TS t+1 to TS *T* is denoted by Y(t), i.e.,

(3) $$Y\left(t\right)≜V^{FE}\left(T\right)-V^{*}\left(t\right).$$

The number of data packets which can be sent by sensor *i* from TS t+1 to TS *T* is denoted by $Y_{i}\left(t\right)$, i.e.,

(4) $$Y_{i}\left(t\right)≜V_{i}^{FE}\left(T\right)-\max_{\pi^{*}\in G^{*}}V_{i}^{*}\left(t\right),$$

where $G^{*}$ is the set of all throughput-optimal policies (under different throughput-optimal policies, the throughput of a sensor *i* in first *t* TSs may be differed since sensor *i* may be scheduled by the FC different times under different throughput-optimal policies).

Notice that $Y\left(0\right)=\sum_{i=1}^{M}\left\lfloor E_{i}\left(T\right)\right\rfloor$ and $Y_{i}\left(0\right)=\left\lfloor E_{i}\left(T\right)\right\rfloor$.

**Definition** **4.** ***Intensity of sensor*** *i, $\rho_{i}$, is defined as the integer part of the total energy harvested by sensor i over the time horizon, T, normalized by $\frac{KT}{M}$, i.e.,*

$$\rho_{i}≜\frac{M\left\lfloor E_{i}\left(T\right)\right\rfloor }{KT}.$$

**Definition** **5.** ***Intensity*** *, ρ, is defined as the sum of integer parts of the total energy harvested by all sensors over the time horizon, T, normalized by KT, i.e.,*

$$\rho ≜\frac{\sum_{i=1}^{M}\left\lfloor E_{i}\left(T\right)\right\rfloor }{KT}=\frac{\sum_{i=1}^{M}\rho_{i}}{M}.$$

**Remark** **1.** If both of the following conditions, $Y_{i}\left(t\right)\leq \left(T-t\right)$∀i∈S, ∀t and Y(t)≤K(T−t)∀t, are satisfied, then an optimal policy becomes a fully efficient policy, i.e., $V^{*}\left(T\right)=V^{FE}\left(T\right)$. Otherwise, an optimal policy cannot achieve throughput of $V^{FE}\left(T\right)=\sum_{i=1}^{M}\left\lfloor E_{i}\left(T\right)\right\rfloor$, i.e., $V^{*}\left(T\right)<V^{FE}\left(T\right)$. In the cases violating at least one of these conditions, comparing a policy with a fully efficient policy is much simpler than comparing it with an optimal policy. Therefore, we also introduce the notion of fully efficient policy.

For ease of reference, our commonly used notation is summarized in Table 1.

## 3. Efficiency of Myopic and Round Robin Policies

A similar problem to the problem at hand is studied in [34,35] for certain specific cases under discounted reward criterion. RR based MP is proposed in both papers in which they prove the optimality of this policy for certain specific cases. We applied this RR based myopic policy to the problem at hand. As the MP in [34,35] is an RR policy with quantum = 1 TS, we investigate only RR policies with quantum = 1 TS, denoted by $\pi^{RR}$, in this paper.

**Definition** **6.** *For the network that consists of M sensors and an FC with K channels, a* ***Round Robin (RR) policy with quantum = 1 TS*** *is an RR policy under which the FC schedules the sensors by allocating one TS to each sensor for data transmission in a period of $\frac{M}{K}$ TSs (Quantum is defined as the number of TSs allocated to each sensor in a period (round) by an RR policy. An RR policy with quantum=n TSs is an RR policy that allocates n TSs to each sensor in a period (round) of $\frac{Mn}{K}$, and so on. For applicability of RR policies with quantum = n≥1 TS, $\frac{M}{K}$ must be an integer).*

In this section, we show that RR policies with quantum = 1 TS are generally suboptimal by Theorem 1. Next, we study their efficiencies more precisely and obtain an upper bound for their efficiencies by Theorem 2. Then, we show that an RR policy with quantum = 1 TS achieves almost the same efficiency as another RR policy with quantum = 1 TS by Theorem 3, which implies that the MP in [34,35] is generally suboptimal and the upper bound obtained for RR policies with quantum = 1 TS is also valid for the MP in [34,35].

**Theorem** **1.** *If there exist some sensors i∈S such that $Y_{i}\left(t\right)>\left\lceil \frac{K(T-t)}{M}\right\rceil$ for some t<T, all RR policies with quantum = 1 TS have efficiency lower than 100% even if fully efficient policy exists for Problem 1 (they are suboptimal).*

**Proof.**  Under an RR policy with quantum = 1 TS, each sensor is visited by the FC either $\left\lfloor \frac{K(T-t)}{M}\right\rfloor$ or $\left\lceil \frac{K(T-t)}{M}\right\rceil$ times if $\frac{K(T-t)}{M}$ is not an integer. If $\frac{K(T-t)}{M}$ is an integer, then FC allocates $\frac{K(T-t)}{M}$ TSs to each sensor. This means that total number of transmissions from any sensor cannot exceed $\left\lceil \frac{K(T-t)}{M}\right\rceil$. $Y_{i}\left(t\right)>\left\lceil \frac{K(T-t)}{M}\right\rceil$ implies that sensor *i* need to send more than $\left\lceil \frac{K(T-t)}{M}\right\rceil$ data packets in TSs t+1 through *T* so as to send $V_{i}^{FE}\left(T\right)=\left\lfloor E_{i}\left(T\right)\right\rfloor$ packets, which must be sent by each sensor for full efficiency. Hence, even if a fully efficient policy exists, any RR policy with quantum = 1 TS is not fully efficient for Problem 1 (they are suboptimal). ☐

### 3.1. Efficiency Bounds of RR Policies with Quantum = 1 TS

In this subsection, efficiency bounds of RR policies with quantum = 1 TS are studied precisely for general EH processes.

**Lemma** **1.** *There exists a class of EH processes with intensity $\rho_{i}$, such that, for these EH processes, some sensor i transmits lower than $\min\left\{Y_{i}\left(0\right),q_{i}\right\}$ data packets over a time horizon, T, by an RR policy with quantum = 1 TS, where $q_{i}$ is the number of TSs allocated to sensor i over the time horizon, i.e., $q_{i}\in \left\{\left\lfloor \frac{KT}{M}\right\rfloor ,\left\lfloor \frac{KT}{M}\right\rfloor +1\right\}$.*

**Proof.**  Please see Appendix A. ☐

Now, we introduce two sets, $H_{1}$ and $H_{2}$, which are used in Theorem 2, Corollaries 1 and 2. $H_{1}$ denotes the index set of the sensors *i* for which $\left\lfloor \frac{KT}{M}\right\rfloor +1$ TSs are allocated and $Y_{i}\left(0\right)>\left\lfloor \frac{KT}{M}\right\rfloor +1$. Moreover, $H_{2}$ denotes the index set of the sensors *i* for which $\left\lfloor \frac{KT}{M}\right\rfloor$ TSs are allocated and $Y_{i}\left(0\right)>\left\lfloor \frac{KT}{M}\right\rfloor$. By definition, $\rho_{i}>1$ for sensors $i\in H≜H_{1}\cup H_{2}$.

**Theorem** **2.** *For general EH processes,*

***(i)***  *If $\frac{KT}{M}\notin Z$, efficiency of an RR policy with quantum=1 TS satisfies*
  $$\eta \left(\pi^{RR}\right)\leq 1-\frac{\sum_{i\in H}\left(\rho_{i}-1\right)-\frac{M}{KT}\left|H_{1}\right|}{M\rho }.$$
***(ii)***  *If $\frac{KT}{M}\in Z$, efficiency of an RR policy with quantum=1 TS satisfies*
  $$\eta \left(\pi^{RR}\right)\leq 1-\frac{\sum_{i\in H}\left(\rho_{i}-1\right)}{M\rho }.$$

**Proof.**  Please see Appendix B. ☐

From Theorem 2, we derive the following corollaries.

**Corollary** **1.** *For sufficiently large T, we have M≪KT. By the definition, $\left|H_{1}\right|<\sum_{i\in H_{1}}\rho_{i}\leq M\rho$ so $\frac{\left|H_{1}\right|}{KT\rho }\ll 1$. Hence, upper bound of efficiency of an RR policy with quantum=1 TS can approximately be written as $1-\frac{\sum_{i\in H}\left(\rho_{i}-1\right)}{M\rho }$.*

**Corollary** **2.** *For sufficiently large T, $\left|H_{1}\right|<M\rho \ll KT\rho$. If $\frac{\left|H_{1}\right|}{KT\rho }$ in part (i) of Theorem 2 is neglected and*

***(i)***  If H=∅, then $\eta (\pi^{RR})\leq 1$.
***(ii)***  If $\rho_{i}=c$, ∀i∈H and $\rho_{i}=0$, ∀i∈S−H, then $\eta \left(\pi^{RR}\right)\leq \min\left\{1,\frac{1}{c}\right\}$ where $c\in R^{+}$.

### 3.2. Throughput Difference of RR Policies with Quantum = 1 TS

We will prove that the throughput difference between any two RR policies with quantum = 1 TS cannot be greater than M−K in a time horizon. Recall that, for all RR policies with quantum = 1 TS, the scheduling is periodic with a period of $\frac{M}{K}$ TSs. The only difference between any two RR policies with quantum = 1 TS is their initial scheduling time, $t_{0}$; therefore, an RR policy with quantum = 1 TS that starts to send first packet in TS $t_{0}$ can be labeled as $RR_{t_{0}}$, where $1\leq t_{0}\leq \frac{M}{K}$.

**Lemma** **2.** *The number of transmissions of sensor i can be varied at most one under two different RR policies with quantum = 1 TS over the time horizon T, i.e.,*

$$\max_{1\leq t_{0}\leq \frac{M}{K}}V_{i}^{RR_{t_{0}}}\left(T\right)-\min_{1\leq t_{0}\leq \frac{M}{K}}V_{i}^{RR_{t_{0}}}\left(T\right)\leq 1.$$

**Proof.**  Please see Appendix C. ☐

The following example is given to illustrate Lemma 2.

**Example** **1.** Assume that T=14, M=3 and K=1. There are three RR policies with quantum=1 TS which schedule the same sensor node i in different TSs as follows.

*$RR_{1}$: Sensor i is scheduled in TSs 1,4,7,10,13,*

*$RR_{2}$: Sensor i is scheduled in TSs 2,5,8,11,14,*

*$RR_{3}$: Sensor i is scheduled in TSs 3,6,9,12.*

*Notice that sensor i have the chance of transmission five times under $RR_{1}$ and $RR_{2}$ while it has a chance of transmission only four times under $RR_{3}$. Denote the transmission times of sensor i under different RR policies by $t_{j}^{n}$ where n=1,2,3 are the indices (initial scheduling times) of RR policies and j=1,…,4 or 5 are transmission times of sensor i under the applied RR policy in this example. $E_{i}\left(t_{j}^{2}\right)\geq E_{i}\left(t_{j}^{1}\right)$ for j=1,…,5 and $E_{i}\left(t_{j+1}^{2}\right)\geq E_{i}\left(t_{j}^{3}\right)$ for j=1,…,4 and $E_{i}\left(t_{j+1}^{1}\right)\geq E_{i}\left(t_{j}^{3}\right)$ for j=1,…,4; therefore, $V_{i}^{RR_{2}}\left(14\right)\geq V_{i}^{RR_{1}}\left(14\right)=V_{i}^{RR_{1}}\left(13\right)\geq V_{i}^{RR_{3}}\left(14\right)=V_{i}^{RR_{3}}\left(12\right)$. On the other hand, $E_{i}\left(t_{j}^{2}\right)\leq E_{i}\left(t_{j}^{3}\right)$ for j=1,…,4; therefore, $V_{i}^{RR_{2}}\left(11\right)\leq V_{i}^{RR_{3}}\left(12\right)$. The difference between the most and the least efficient RR policies with quantum = 1 TS are*

$$\begin{array}{cc} V_{i}^{RR_{2}}\left(14\right)-V_{i}^{RR_{3}}\left(14\right) & =V_{i}^{RR_{2}}\left(14\right)-V_{i}^{RR_{3}}\left(12\right)\\ & \leq V_{i}^{RR_{2}}\left(14\right)-V_{i}^{RR_{2}}\left(11\right)\\ & \leq 1. \end{array}$$

*Hence, it is observed that throughput of a sensor i can be varied at most 1 under any RR policies with quantum = 1 TS.*

The following theorem is based on the extension of Lemma 2 for the whole network.

**Theorem** **3.** *An RR policy with quantum = 1 TS achieves at most M−K more throughput than another RR policy with quantum = 1 TS over the time horizon T, i.e.,*

$$\max_{\pi^{RR}\in G^{RR}}V^{RR}\left(T\right)-\min_{\pi^{RR}\in G^{RR}}V^{RR}\left(T\right)\leq M-K,$$

*where $G^{RR}$ is the set of all RR policies with quantum = 1 TS.*

**Proof.**  In this proof, we first consider the case of transmitting messages of $\frac{M}{K}$ sensors over a single channel under an RR policy with quantum = 1 TS. Then, we extend the result of Lemma 2 to the case of multiple (*K*) channels. Notice that

$$t_{0}=mod_{\left(\frac{M}{K}\right)}TI_{\left\{mod_{\left(\frac{M}{K}\right)}T\neq 0\right\}}+\frac{M}{K}I_{\left\{mod_{\left(\frac{M}{K}\right)}T=0\right\}}$$

is the best choice regardless of the sensor *i* and this most efficient RR policy must be applied to one of the $\frac{M}{K}$ sensors over a single channel. When *K* channels of the FC are considered, this most efficient RR policy must be applied to *K* of *M* sensors. By considering this fact and Lemma 2, an RR policy with quantum = 1 TS transmits at most M−K more data packets than another RR policy with quantum = 1 TS. ☐

**Remark** **2.** *From Theorem 3 and Definition 2,*

$$\max_{\pi^{RR}\in G^{RR}}\eta \left(\pi^{RR}\right)-\min_{\pi^{RR}\in G^{RR}}\eta \left(\pi^{RR}\right)\leq \frac{M-K}{V^{FE}\left(T\right)}=\frac{M-K}{\rho KT},$$

*where $G^{RR}$ is the set of all RR policies with quantum = 1 TS.*

As the MP in [34,35] is also an RR policy with quantum = 1 TS, from Remark 2, it has almost same efficiency as another RR policy with quantum=1 TS for sufficiently large time horizon *T*, i.e., $\eta \left(\pi^{MP}\right)\approx \eta \left(\pi^{RR}\right)$ if K,M≪T. For sufficiently large time horizons, these results can be extended for RR policies with quantum = n>1 TSs.

## 4. Numerical Results

In this section, efficiency of the myopic policy (MP) is evaluated for the cases of infinite battery and finite battery with B=50 (B=50 implies that the battery of a sensor can store energy enough to send 50 data packets since we assume that each data packet transmission requires one unit of energy) at the time horizons varying from 0 to 2000 TSs via simulations (as efficiency of a policy are defined only for the time horizon, *T*, we obtain efficiency vs. time horizon figures in this section. Notice that efficiency of the MP at T=0 is taken as 0 for these simulations).

For each node *i*, the Markovian EH process is modelled by a state space $\left\{0,1,2\right\}$ and a 3×3 transition matrix $\left[\begin{array}{ccc} 0.90 & 0.05 & 0.05\\ 0.05 & 0.90 & 0.05\\ 0.05 & 0.05 & 0.90 \end{array}\right]$. The harvested energy for sensor *i* in TS *t* is $E_{i}^{h}\left(t\right)=\frac{K}{M}\times \rho_{i}\times M_{i}\left(t\right)$ where EH state of sensor *i* in TS *t* is denoted by $M_{i}\left(t\right)\in \left\{0,1,2\right\}$.

Notice that efficiency of the MP in [34,35] is almost the same as efficiency of an RR policy with quantum=1 TS since $\eta \left(\pi^{RR}\right)\approx \eta \left(\pi^{MP}\right)$ for sufficiently large *T* from Theorem 3 and Remark 2. We observe efficiency of the MP under nonuniform EH processes (it is obvious that the MP achieve efficiencies close to efficiency of an optimal policy for uniform EH processes. As this case is trivial, we did not show the results for this case).

The simulations are made by taking M=100 and K=10 under Markovian and i.i.d EH processes with various intensities which are adjusted by choosing intensities of some sensors as 3.0 and choosing others as 0.3 as explained in Table 2 (In WSNs, it is highly possible that some EH sensors can harvest energy much efficiently than others due to their energy harvesting resource (solar, piezoelectric, RF, wind, etc.) and environmental conditions. For example, solar energy harvesting is generally more efficient than the others. Therefore, we choose intensities of some sensors much larger than the others (3.0 for some sensors and 0.3 for the remaining ones) in order to represent the difference between the amount of energy harvested by sensors).

### 4.1. Infinite Capacity Battery

**Figure 2:**
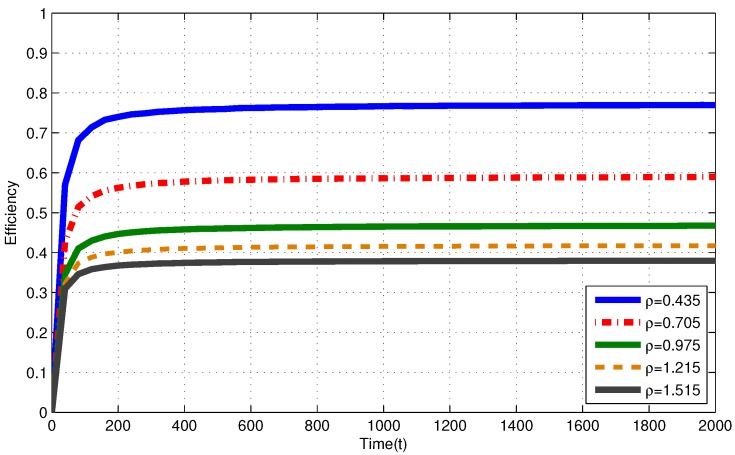
Efficiency of the myopic policy (MP) for i.i.d. EH processes under infinite capacity battery assumption.

**Figure 3:**
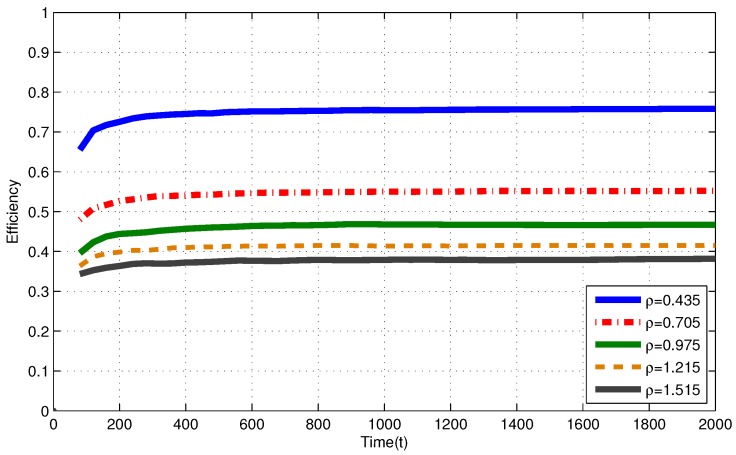
Efficiency of the myopic policy (MP) for Markov EH processes under infinite capacity battery assumption.

In Figure 2, for i.i.d. EH process with ρ≤1, efficiencies of the MP at T=2000 are 0.758, 0.564 and 0.469 for ρ=0.435, ρ=0.705 and ρ=0.975, respectively. In Figure 3, for Markov EH process with ρ≤1, at T=2000, the MP achieves efficiency of 0.758, 0.552 and 0.467 for ρ=0.435, ρ=0.705 and ρ=0.975, respectively. The dramatic difference between efficiencies of the MP in these three intensities is expected since Theorem 2 and Corollary 1 state that, as the number of nodes with intensity $\rho_{i}>1$ increases, the efficiency of RR policies with quantum = 1 TS decrease. Notice that, from Theorem 2 and Corollary 1, efficiencies of an RR policy with quantum = 1 TS at T=2000 are expected to be $\eta (\pi^{RR})\leq 0.770$, $\eta (\pi^{RR})\leq 0.574$ and $\eta (\pi^{RR})\leq 0.487$ for ρ=0.435, ρ=0.705 and ρ=0.975, respectively. When EH processes have memory (Markov processes), we observe similar results to the results in memoriless (i.i.d.) EH processes with same intensity.

In Figure 2, for i.i.d. EH process with ρ>1, efficiencies of the MP at T=2000 are 0.417 and 0.380 for ρ=1.245 and ρ=1.515, respectively. In Figure 3, for Markov EH process with ρ>1, at T=2000, the MP achieves efficiency of 0.415 and 0.381 for ρ=1.245 and ρ=1.515, respectively. From Corollary 1, efficiencies of an RR policy with quantum = 1 TS at T=2000 are expected to be $\eta (\pi^{RR})\leq 0.438$ and $\eta (\pi^{RR})\leq 0.406$ for ρ=1.245 and ρ=1.515, respectively. For EH processes with ρ>1, from Definitions 1–3, efficiency of an optimal policy is $\eta \left(\pi^{*}\right)=\frac{V^{*}\left(T\right)}{V^{FE}\left(T\right)}\leq \frac{1}{\rho }.$ Hence, at T=2000, $\eta (\pi^{*})\leq 0.803$ and $\eta (\pi^{*})\leq 0.660$ for ρ=1.245 and ρ=1.515, respectively. Markov EH processes have similar results to the results for i.i.d. EH processes with same intensity.

### 4.2. Finite Capacity Battery

In this subsection, the simulations are made by considering a finite battery capacity with B=50 under Markovian and i.i.d EH processes with the intensities in Table 2.

**Figure 4:**
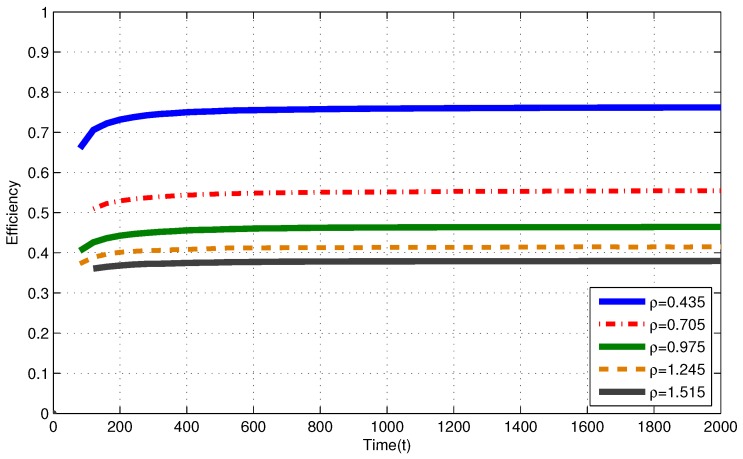
Efficiency of the myopic policy (MP) for Markov EH processes under finite capacity ($B_{i}=50$) battery assumption.

**Figure 5:**
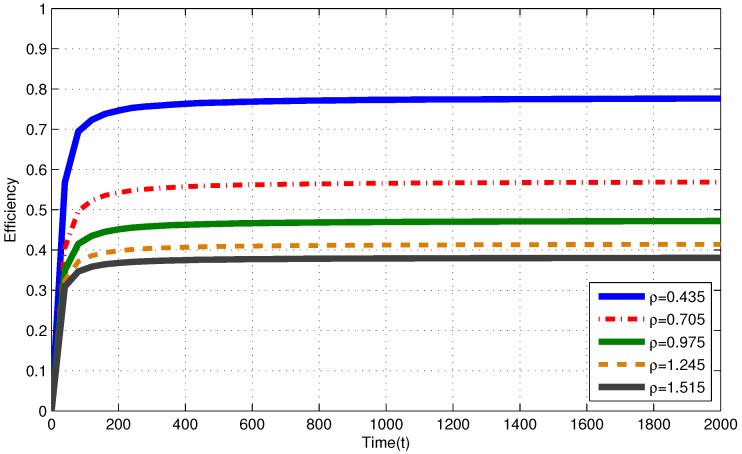
Efficiency of the myopic policy (MP) for i.i.d. EH processes finite capacity ($B_{i}=50$) battery assumption.

In Figure 4 (i.i.d. EH process), efficiencies of the MP at T=2000 are 0.757, 0.555, 0.464, 0.415 and 0.380 for ρ=0.435, ρ=0.705, ρ=0.975, ρ=1.245 and ρ=1.515, respectively. In Figure 5 (Markov EH process), at T=2000, the MP achieves efficiency of 0.756, 0.564, 0.462, 0.414 and 0.380 for ρ=0.435, ρ=0.705, ρ=0.975, ρ=1.245 and ρ=1.515, respectively. From Corollary 1, efficiencies of an RR policy with quantum = 1 TS at T=2000 are expected to be $\eta (\pi^{RR})\leq 0.758$, $\eta (\pi^{RR})\leq 0.564$, $\eta (\pi^{RR})\leq 0.469$$\eta (\pi^{RR})\leq 0.438$ and $\eta (\pi^{RR})\leq 0.406$ for ρ=0.435, ρ=0.705, ρ=0.975, ρ=1.245 and ρ=1.515, respectively.

Markov EH processes have similar results to the results for i.i.d. EH processes with the same intensity.

### 4.3. Discussion

In this subsection, the efficiencies of the myopic policy in both infinite and finite capacity battery cases are compared with each other based on the numerical results in Table 3. Besides this, these numerical results are compared with the expected upper bounds for efficiency of myopic policy. Finally, the complexity of the Round-Robin based myopic policy is investigated.

From Table 3, it can observed that the maximum efficiency difference (0.009) occurs between B=∞ (0.564) and B=50 (0.555) for i.i.d. EH processes with intensity ρ=0.705. For this intensity, the efficiency in finite battery case is 1.596% less than the efficiency in infinite battery case. Besides this, the minimum difference (0.000) occurs between B=∞ (0.380) and B=50 (0.380) for i.i.d. EH processes with ρ=1.515. Therefore, the efficiency of MP for B=50 is only 1.596% less than that for B=∞ at most. Hence, we can conclude that the MP can achieve almost the same throughput performance with a reasonable finite capacity (B=50) battery as that with infinite capacity battery.

Moreover, from Table 3, it can observed that the maximum deviation (difference) occurs between the upper bound (0.406) and efficiency of MP for i.i.d. EH processes (0.380) with intensity ρ=1.515. For this intensity, the efficiency is 6.40% less than the upper bound. Besides this, the minimum deviation occurs between the upper bound (0.770) and efficiency of MP for i.i.d. EH processes (0.758) with ρ=0.435. For this intensity, the efficiency is only 1.56% less than the upper bound. Based on these results, we can say that the upper bounds for efficiency of the MP are generally tight.

In addition, the Round Robin based myopic policy is a simple policy for this problem. There is an initial ordering and the order is kept during the time interval when Round Robin based scheduling is performed. Sorting algorithms that are required for initial ordering has a worst case complexity of $O(M^{2})$ and Round Robin algorithm has a complexity of O(1). Therefore, the myopic policy is a low-complexity solution for the problem at hand.

## 5. Conclusions

This paper investigates a problem occurring in a single-hop WSN where an FC schedules a set of EH sensors to collect data from them. The FC does not know the instantaneous battery states or the statistics of EH processes at sensors that are data backlogged and the communication is error-free. There is no leakage from the infinite-capacity batteries. The problem at hand is set up as an average throughput (reward) maximization problem. The myopic policy in [34,35] that has an RR structure is applied to this problem as a solution. It is shown that RR policies with quantum = 1 TS are suboptimal for a broad class of EH processes. Next, an upper bound is obtained for efficiencies of RR policies with quantum = 1 TS. Then, it is shown that the myopic policy have almost equal efficiency as another RR policy with quantum = 1 TS. Furthermore, numerical results show that the myopic policy is suboptimal for a broad class of EH processes although it achieves optimality for certain specific cases.

As a future work, we search for a simple, optimal solution to this problem for quite general EH processes. As another future work, we look for extending the single hop problem to multi hop case. Moreover, we plan to investigate the same problem under finite capacity battery case and make the throughput performance analysis of the myopic policy under finite capacity battery case. In addition, we will work to extend the problem in our future works such that we can consider the network lifetime as a performance metric. We believe that novel approaches and concepts in this paper will give insight to the researchers who study similar scheduling problems.

## Figures and Tables

**Table 1:** Summary of commonly used symbols and notation.

Symbol	Definition
*M*	The number of energy harvesting nodes
*K*	The number of mutually orthogonal channels of FC
*S*	The index set of all nodes
*T*	The time horizon
$V^{\pi }\left(t\right)$	Throughput of all nodes in TSs 1 through *t* under a policy π
$V_{i}^{\pi }\left(t\right)$	Throughput of node *i* in TSs 1 through *t* under a policy π
η(π)	Efficiency of a policy π
$Y_{i}\left(t\right)$	The number of packets which can be sent by node *i* in (t,T]
$\rho_{i}$	Intensity of node *i*
ρ	Intensity

**Table 2:** *W* and *L* denote the numbers of sensors with $\rho_{i}=0.3$ and $\rho_{i}=3.0$, respectively. ρ denotes the resultant intensity.

W	95	85	75	65	55
L	5	15	25	35	45
ρ	0.435	0.705	0.975	1.245	1.515

**Table 3:** Efficiency of MP for IID and Markov EH processes under both infinite and finite capacity battery assumptions B=∞ and B=50 stands for infinite and finite capacity batteries, respectively. ρ denotes the intensity. Max. efficiency difference between B=∞ and B=50 represents the efficiency difference between B=∞ and B=50 cases for the same intensity. Max. efficiency difference (%) btw. B=∞ and B=50 represents the percentage of efficiency difference between B=∞ and B=50 cases over the efficiency in B=∞ case for the same intensity. Max. deviation between the bound and efficiency of MP represents the difference between the upper bound of efficiency of MP and minimum efficiency result of MP for the same intensity.

ρ	0.435	0.705	0.975	1.245	1.515
Efficiency of MP for Markov EH process, B=∞	0.758	0.562	0.467	0.415	0.381
Efficiency of MP for Markov EH process, B=50	0.756	0.561	0.462	0.414	0.380
Efficiency of MP for IID EH process, B=∞	0.758	0.564	0.469	0.417	0.380
Efficiency of MP for IID EH process, B=50	0.757	0.555	0.464	0.415	0.380
Max. efficiency difference between B=∞ and B=50	0.002	0.009	0.005	0.002	0.001
Max. efficiency difference (%) btw. B=∞ and B=50	0.264	1.596	1.071	0.480	0.262
Upper bound for efficiency of MP	0.770	0.574	0.487	0.438	0.406
Max. deviation between the bound and efficiency of MP	0.014	0.019	0.025	0.024	0.026

## Abbreviations

The following abbreviations are used in this manuscript:

IoT	Internet of Things
WSN	Wireless sensor network
EH	Energy harvesting
FC	Fusion center
TS	time slot
SNR	Signal to Noise Ratio
RF	Radio frequency
POMDP	Partially Observable Markov Decision Processes
DP	Dynamic Programming
RMAB	Restless Multi Armed Bandit
PSPACE	Polynomial Space
MP	Myopic policy
RR	Round-Robin
IID	independent and identically distributed

## Appendix A. Proof of Lemma 1

Define a new function, $\rho_{i}\left(t\right)$, for a sensor *i* as

(A1) $$\rho_{i}\left(t\right)≜\frac{V_{i}^{FE}\left(T\right)-\max_{\pi^{RR}\in G^{RR}}V_{i}^{RR}\left(t\right)}{\left\lceil \frac{K(T-t)}{M}\right\rceil },$$

where $G^{RR}$ is the set of all RR policies with quantum = 1 TS.

Assume $\rho_{i}\left(t\right)>1>\rho_{i}$ for some sensor *i* and *t*. From Label (A1),

(A2) $$\begin{array}{ccc} \frac{V_{i}^{FE}\left(T\right)-\max_{\pi^{RR}\in G^{RR}}V_{i}^{RR}\left(t\right)}{\left\lceil \frac{K(T-t)}{M}\right\rceil } & > & 1,\;\tau \leq t<T,\\ V_{i}^{FE}\left(T\right)-\max_{\pi^{RR}\in G^{RR}}V_{i}^{RR}\left(t\right) & > & \left\lceil \frac{K(T-t)}{M}\right\rceil ,\tau \leq t<T, \end{array}$$

where $\tau =\min_{\rho_{i}\left(t\right)>1}t$.

In the interval, τ≤t<T, sensor *i* can send at most $\left\lceil \frac{K(T-t)}{M}\right\rceil$ data packets under an RR policy with quantum = 1 TS. Therefore, Label (A2) implies that $\min\left\{Y_{i}\left(0\right),q_{i}\right\}=Y_{i}\left(0\right)$ packets cannot be sent by sensor *i* over a time horizon, *T*, where $q_{i}\in \left\{\left\lfloor \frac{KT}{M}\right\rfloor ,\left\lfloor \frac{KT}{M}\right\rfloor +1\right\}$. If the EH process at sensor *i* were a constant EH process with intensity $\rho_{i}<1$ (for these EH processes, $\rho_{i}\left(t\right)=\rho_{i}$ for all 1≤t<T), then sensor *i* would send $\min\left\{Y_{i}\left(0\right),q_{i}\right\}=Y_{i}\left(0\right)$ packets over a time horizon, *T*, from Definition 5 and Remark 1. Hence, efficiencies of RR policies with quantum=1 TS under such an EH process that $\rho_{i}\left(t\right)>1>\rho_{i}$ for some sensor *i* and *t* become lower than they do under a constant EH process with intensity $\rho_{i}$. This implies that there exists a class of EH processes for which some sensor *i* cannot achieve the throughput of $\min\left\{Y_{i}\left(0\right),q_{i}\right\}$ packets over time horizon, *T*.

## Appendix B. Proof of Theorem 2

Define

(A3) $$q≜\frac{KT}{M}-\frac{m}{M},$$

where *q* and *m* are integers and 0≤m≤M−K. Recall $\frac{M}{K}\in Z$ for applicability of RR policies with quantum = 1 TS.

**Case i:** Assume $\frac{KT}{M}\notin Z$ and the RR policy with quantum = 1 TS starts to scheduling with sensor 1 without loss of generality. Notice that K≤m≤M−K if $\frac{KT}{M}\notin Z$. If the order 1,2,…,M is followed by the RR policy with quantum=1 TS for the scheduling, sensors 1,…,m are scheduled q+1 times and sensors m+1,…,M are scheduled *q* times over a time horizon, *T*. From Lemma 1, for some EH processes,

(A4) $$\;V^{RR}\left(T\right)\;<\;\;\sum_{i=1}^{m}\min\left\{Y_{i}\left(0\right),q+1\right\}+\;\;\sum_{i=m+1}^{M}\;\;\min\left\{Y_{i}\left(0\right),q\right\};$$

otherwise, Label (A4) becomes equality for other EH processes. Hence,

(A5) $$\;V^{RR}\left(T\right)\;\leq \;\;\sum_{i=1}^{r}\min\left\{Y_{i}\left(0\right),q+1\right\}+\;\;\sum_{i=r+1}^{M}\;\;\min\left\{Y_{i}\left(0\right),q\right\}.$$

Let $S=H_{1}\cup H_{2}\cup \left(S-H_{1}-H_{2}\right),$ where $H_{1}\subset \left\{1,\ldots ,m\right\}$ and $H_{2}\subset \left\{m+1,\ldots ,M\right\}$ are the index sets of sensors that have enough energy to send more than q+1 and *q* data packets, respectively. With this specification, the total throughput is

(A6) $$V^{RR}\left(T\right)\leq \sum_{i\in H_{1}}\left(q+1\right)+\sum_{i\in H_{2}}q+\sum_{i\in S-(H_{1}\cup H_{2})}Y_{i}\left(0\right).$$

From Label (A6) and Definition 3,

(A7) $$\begin{array}{ccc} \eta (\pi^{RR})\;\;\;\; & = & \;\;\;\;\frac{V^{RR}\left(T\right)}{Y(0)}\\ & \leq & \;\;\;\;\frac{\sum_{i\in H_{1}}\;\left(q+1\right)+\sum_{i\in H_{2}}q+\sum_{i\in S}Y_{i}\left(0\right)-\;\;\sum_{i\in H_{1}\cup H_{2}}\;\;\;Y_{i}\left(0\right)}{\sum_{i\in S}Y_{i}\left(0\right)}\\ & = & \;\;\;\;1-\frac{\sum_{i\in H_{1}\cup H_{2}}\;Y_{i}\left(0\right)-\sum_{i\in H_{1}}\left(q+1\right)-\sum_{i\in H_{2}}q}{\sum_{i\in S}Y_{i}\left(0\right)}. \end{array}$$

If the numerator and denominator of the second term of the right-hand side in Label (A7) are normalized by $\frac{KT}{M}$,

(A8) $$\eta \left(\pi^{RR}\right)\;\leq \;1-\frac{\sum_{i\in H_{1}\cup H_{2}}\;\;\;\;\frac{MY_{i}\left(0\right)}{KT}-\;\sum_{i\in H_{1}}\;\;\frac{M\left(q+1\right)}{KT}-\;\sum_{i\in H_{2}}\;\;\frac{Mq}{KT}}{\sum_{i\in S}\frac{MY_{i}\left(0\right)}{KT}}.$$

From Definition 4, $\rho_{i}=\frac{MY_{i}\left(0\right)}{KT}$. Labels (A3) and (A8) yields

(A9) $$\eta \left(\pi^{RR}\right)\leq 1-\frac{A}{\sum_{i\in S}\rho_{i}},$$

where

$$\begin{array}{ccc} A\;\;\; & = & \;\;\;\;\sum_{i\in H_{1}\cup H_{2}}\rho_{i}-\sum_{i\in H_{1}}\left(1+\frac{M-m}{KT}\right)-\sum_{i\in H_{2}}\left(1-\frac{m}{KT}\right)\\ \;\; & = & \;\;\;\;\sum_{i\in H_{1}\cup H_{2}}\;\left(\rho_{i}-1\right)-\sum_{i\in H_{1}}\frac{M}{KT}+\;\sum_{i\in H_{1}\cup H_{2}}\frac{m}{KT}\\ & \geq & \;\;\;\;\sum_{i\in H_{1}\cup H_{2}}\;\left(\rho_{i}-1\right)-\frac{M}{KT}\left|H_{1}\right|. \end{array}$$

Hence, efficiencies of RR policies with quantum = 1 TS satisfy

$$\eta \left(\pi^{RR}\right)\leq 1-\frac{\sum_{i\in H}\left(\rho_{i}-1\right)-\frac{M}{KT}\left|H_{1}\right|}{M\rho }.$$

**Case ii:** If $\frac{KT}{M}\in Z$, then $q=\frac{KT}{M}$ since m=0 in Label (A3) in this case. Let $S=H_{2}\cup \left(S-H_{2}\right)$ where $H_{2}$ is index set of sensors that have enough energy to send more than *q* data packets. (Notice that $H_{1}=\emptyset$ and $H=H_{1}\cup H_{2}=H_{2}$ if $\frac{KT}{M}\in Z$.) By following similar steps in part (i), we obtain

(A10) $$\begin{array}{ccc} V^{RR}\left(T\right) & \leq & \sum_{i=1}^{M}\min\left\{Y_{i}\left(0\right),q\right\}\\ & \leq & \sum_{i\in H_{2}}q+\sum_{i\in S-H_{2}}Y_{i}\left(0\right). \end{array}$$

From Label (A10) and Definition 3, efficiency of RR policies with quantum = 1 TS can be expressed as

(A11) $$\begin{array}{ccc} \eta (\pi^{RR})\;\;\;\; & = & \;\;\;\;\frac{V^{RR}\left(T\right)}{Y(0)}\\ & = & \frac{\sum_{i\in H_{1}}\left(\sigma +1\right)+\sum_{i\in H_{2}}\sigma -\sum_{i\in H_{1}\cup H_{2}}W_{i}\left(0\right)+\sum_{i\in H_{1}\cup H_{2}}W_{i}\left(0\right)+\sum_{i\in S-H_{1}-H_{2}}W_{i}\left(0\right)}{\sum_{i\in S}W_{i}\left(0\right)}\\ & \leq & \;\;\;\;\frac{\sum_{i\in H_{1}\cup H_{2}}q+\sum_{i\in S}Y_{i}\left(0\right)-\;\;\sum_{i\in H_{1}\cup H_{2}}\;\;\;Y_{i}\left(0\right)}{\sum_{i\in S}Y_{i}\left(0\right)}\\ & \leq & \;\;\;\;1-\frac{\sum_{i\in H_{2}}\frac{MY_{i}\left(0\right)}{KT}-\sum_{i\in H_{2}}\frac{Mq}{KT}}{\sum_{i\in S}\frac{MY_{i}\left(0\right)}{KT}}. \end{array}$$

From Label (A3), Definitions 4 and 5, we obtain

$$\eta \left(\pi^{RR}\right)\leq 1-\frac{\sum_{i\in H}\left(\rho_{i}-1\right)}{M\rho }.$$

## Appendix C. Proof of Lemma 2

Recall that $\frac{M}{K}\in Z$ for applicability of RR policies with quantum = 1 TS. There are $\frac{M}{K}$ RR policies with quantum=1 TS which start to schedule a sensor *i* in different TSs. Recall that $1\leq t_{0}\leq \frac{M}{K}$ where $t_{0}$ is initial time for $RR_{t_{0}}$ policy to schedule the sensor *i*. The proof is divided into two cases:

**Case i:** Assume that $\frac{KT}{M}\notin Z$. Then, $\frac{KT}{M}≜q+\frac{Kr}{M}$ where *q* and *r* are integers such that $1\leq r\leq \frac{M}{K}-1$ and $\frac{M}{K}\in Z$. Some of the RR policies with quantum=1 TS schedules a sensor *i* q+1 times, whereas the other RR policies schedules the sensor *i**q* times. The set of initial times of the RR policies which scheduled sensor *i* q+1 times is denoted by $P_{q+1}^{RR}=\left\{1,\ldots ,r\right\},$ whereas the set of others is denoted by $P_{q}^{RR}=\left\{r+1,\ldots ,\frac{M}{K}\right\}$. $t_{j}^{n}$ denotes the $j^{th}$ transmission time of sensor *i* under an RR policy, $RR_{n}$, which starts to schedule sensor *i* in TS *n*, where j=1,…,q or q+1 are transmission times of sensor *i* under the applied RR policy, $RR_{n}$. By definition,

$$E_{i}\left(t_{j+1}^{a}\right)\geq E_{i}\left(t_{j}^{b}\right),\forall a\in P_{q+1}^{RR},\forall b\in P_{q}^{RR},\forall j\in \left\{1,\ldots ,q\right\},$$

which yields

(A12) $$V_{i}^{RR_{a}}\left(T\right)\geq V_{i}^{RR_{b}}\left(T\right),\;\forall a\in P_{q+1}^{RR},\forall b\in P_{q}^{RR}.$$

Notice that $r=mod_{\left(\frac{M}{K}\right)}T\in P_{q+1}^{RR}$ (in fact, $r=\max_{n\in P_{q+1}^{RR}}n$). Therefore,

$$E_{i}\left(t_{j}^{r}\right)\geq E_{i}\left(t_{j}^{a}\right),\;\forall a\in P_{q+1}^{RR},\forall j\in \left\{1,\ldots ,q+1\right\},$$

which yields

(A13) $$V_{i}^{RR_{r}}\left(T\right)\geq V_{i}^{RR_{a}}\left(T\right),\;\forall a\in P_{q+1}^{RR}.$$

Similarly, $r+1=\min_{n\in P_{q}^{RR}}n$. Therefore,

$$E_{i}\left(t_{j}^{r+1}\right)\leq E_{i}\left(t_{j}^{b}\right),\;\forall b\in P_{q}^{RR},\forall j\in \left\{1,\ldots ,q\right\},$$

which yields

(A14) $$V_{i}^{RR_{r+1}}\left(T\right)\leq V_{i}^{RR_{b}}\left(T\right),\;\forall b\in P_{q}^{RR}.$$

From Labels (A12)–(A14),

(A15) $$\begin{array}{ccc} V_{i}^{RR_{r}}\left(T\right) & \geq & V_{i}^{RR_{a}}\left(T\right)\geq V_{i}^{RR_{b}}\left(T\right)\geq V_{i}^{RR_{r+1}}\left(T\right),\\ \forall a & \in & P_{q+1}^{RR},\forall b\in P_{q}^{RR}. \end{array}$$

Furthermore,

$$E_{i}\left(t_{j}^{r+1}\right)\geq E_{i}\left(t_{j}^{r}\right),\forall j\in \left\{1,\ldots ,q\right\},$$

which yields

(A16) $$V_{i}^{RR_{r+1}}\left(t_{j}^{r+1}\right)\geq V_{i}^{RR_{r}}\left(t_{j}^{r}\right),\forall j\in \left\{1,\ldots ,q\right\}.$$

From Label (A16),

(A17) $$\begin{array}{ccc} V_{i}^{RR_{r+1}}\left(T\right) & = & V_{i}^{RR_{r+1}}\left(T-\frac{M}{K}+1\right)\\ & \geq & V_{i}^{RR_{r}}\left(T-\frac{M}{K}\right). \end{array}$$

From Labels (A15) and (A17),

(A18) $$\begin{array}{ccc} \max_{n}V_{i}^{RR_{n}}\left(T\right)-\min_{n}V_{i}^{RR_{n}}\left(T\right) & = & V_{i}^{RR_{r}}\left(T\right)-V_{i}^{RR_{r+1}}\left(T\right)\\ & \leq & V_{i}^{RR_{r}}\left(T\right)-V_{i}^{RR_{r}}\left(T-\frac{M}{K}\right)\\ & \leq & 1. \end{array}$$

Hence, the lemma is proved for the first case, $\frac{KT}{M}\notin Z$.

**Case ii:** If $\frac{KT}{M}\in Z$, each of $\frac{M}{K}$ RR policies with quantum=1 TS schedules a sensor *i* $q=\frac{KT}{M}$ times. The set of initial times of RR policies with quantum=1 TS is denoted by $P_{q}^{RR}=\left\{1,2,\ldots ,\frac{M}{K}\right\}$. $t_{j}^{n}$ denotes transmission time of sensor *i* under an RR policy, $RR_{n}$, where j=1,…,q are transmission times of sensor *i* under the applied RR policy, $RR_{n}$, in this case

$$E_{i}\left(t_{j}^{\frac{M}{K}}\right)\geq E_{i}\left(t_{j}^{n}\right)\geq E_{i}\left(t_{j}^{1}\right),\forall n\in P_{q}^{RR},\forall j\in \left\{1,\ldots ,q\right\},$$

which yields

(A19) $$V_{i}^{RR_{\frac{M}{K}}}\left(T\right)\geq V_{i}^{RR_{n}}\left(T\right)\geq V_{i}^{RR_{1}}\left(T\right),\;\forall n\in P_{q}^{RR}.$$

Moreover,

$$E_{i}\left(t_{j-1}^{\frac{M}{K}}\right)\leq E_{i}\left(t_{j}^{n}\right),\;\forall n\in P_{q}^{RR},\forall j\in \left\{2,\ldots ,q\right\},$$

which yields

(A20) $$V_{i}^{RR_{\frac{M}{K}}}\left(T-\frac{M}{K}\right)\leq V_{i}^{RR_{n}}\left(T\right),\;\forall n\in P_{q}^{RR}.$$

From Labels (A19) and (A20),

(A21) $$\begin{array}{ccc} \max_{n}\;V_{i}^{RR_{n}}\left(T\right)\;\;-\;\min_{n}\;V_{i}^{RR_{n}}\left(T\right)\;\;\;\;\; & = & \;\;\;\;\;V_{i}^{RR_{\frac{M}{K}}}\left(T\right)\;-\;V_{i}^{RR_{1}}\left(T\right)\\ & \leq & \;\;\;\;\;V_{i}^{RR_{\frac{M}{K}}}\;\left(T\right)\;-\;V_{i}^{RR_{\frac{M}{K}}}\;\;\;\left(\;T\;-\;\frac{M}{K}\right)\\ & \leq & \;\;\;\;\;1. \end{array}$$

Hence, the lemma is proved for the second case, $\frac{KT}{M}\in Z$.
